# Questioning emissions-based approaches for the definition of REDD+ deforestation baselines in high forest cover/low deforestation countries

**DOI:** 10.1186/s13021-018-0109-1

**Published:** 2018-10-30

**Authors:** Camille Dezécache, Jean-Michel Salles, Bruno Hérault

**Affiliations:** 1 UMR EcoFoG (AgroParistech, CNRS, Cirad, Inra, Université des Antilles, Université de la Guyane), Université de la Guyane, Campus agronomique de Kourou, 97310 Kourou, French Guiana France; 2 UMR LAMETA (CNRS, Inra, SupAgro, Université de Montpellier), CNRS, Campus Inra-SupAgro, Bat.26, 2 Place Viala, 34060 Montpellier Cedex 2, France; 3 Cirad, UR Forests & Societies, Univ Montpellier, Montpellier, France; 4 Institut National Félix Houphouet-Boigny (INP-HB), Yamoussoukro, Ivory Coast

**Keywords:** Deforestation, Guiana Shield, HFLD countries, Spatial modelling, REDD+, Reference level, Baseline

## Abstract

**Background:**

REDD+ is being questioned by the particular status of High Forest/Low Deforestation countries. Indeed, the formulation of reference levels is made difficult by the confrontation of low historical deforestation records with the forest transition theory on the one hand. On the other hand, those countries might formulate incredibly high deforestation scenarios to ensure large payments even in case of inaction.

**Results:**

Using a wide range of scenarios within the Guiana Shield, from methods involving basic assumptions made from past deforestation, to explicit modelling of deforestation using relevant socio-economic variables at the regional scale, we show that the most common methodologies predict huge increases in deforestation, unlikely to happen given the existing socio-economic situation. More importantly, it is unlikely that funds provided under most of these scenarios could compensate for the total cost of avoided deforestation in the region, including social and economic costs.

**Conclusion:**

This study suggests that a useful and efficient international mechanism should really focus on removing the underlying political and socio-economic forces of deforestation rather than on hypothetical result-based payments estimated from very questionable reference levels.

**Electronic supplementary material:**

The online version of this article (10.1186/s13021-018-0109-1) contains supplementary material, which is available to authorized users.

## Background

Land-use change is a major driver of carbon emissions in tropical regions, whose contribution has been estimated at 9% of the global carbon budget over the last decade, with tropical deforestation being alone the largest contributor to these carbon fluxes [1]. Facing the threat of global warming, reducing carbon emissions from tropical forested areas appears as a necessary option in the first place on the political agenda [2]. Within international mechanisms such as REDD+ , avoided deforestation has emerged as a presumably low-cost option for meeting decrease in carbon emissions requirements [3, 4], mostly because a lot of deforestation was occurring within marginally profitable areas such as shifting cultivation areas [2].

Additionality, i.e. the fact that a decrease in deforestation would not have occurred without efforts made by the country/institution involved, is one of the corner-stones of REDD+ [5] by ensuring that policy changes brought a net decrease in expected deforestation. Formulating a baseline against which carbon credits are evaluated is the most critical component of the REDD+ mechanism [6]. Indeed, in theory, credited emissions must be closely linked with actual emissions reductions. On the contrary, the misformulation of baselines can affect additionality and thus the efficiency of the mechanism [7]. However, reference scenarios are often built upon counterfactual hypotheses which are by nature uncertain [8].

**Fig. 1:**
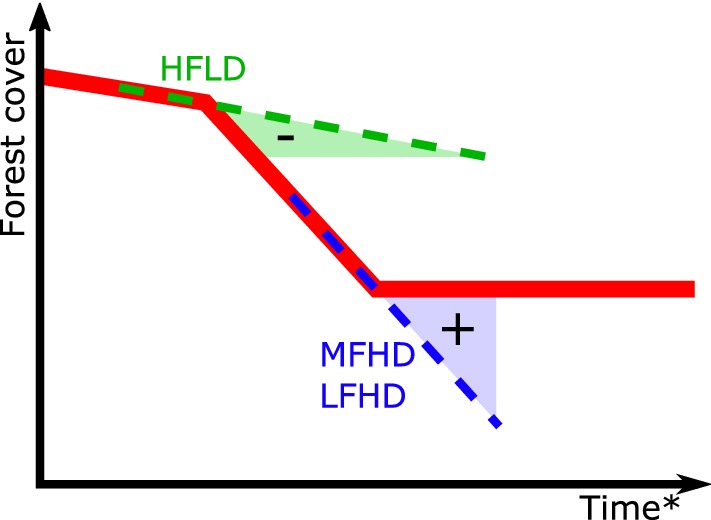
Comparison between two hypothetical REDD+ projects, using a strictly historical reference scenario, in light of the forest transition theory, after Angelsen [54]. The forest transition pathway is displayed in red, with high initial forest cover decreasing ever more rapidly, before stabilizing when forested areas become scarce. Under the Medium Forest/High Deforestation (MFHD) or Low Forest/High Deforestation (LFHD) scenario, where the country would reach the end of the forest transition, the historical baseline (blue dashed line) largely overestimates deforestation, artificially provoking a large amount of credited carbon. On the contrary, under the High Forest/Low Deforestation (HFLD) scenario, the historical baseline (green dashed line) largely underestimates deforestation, inducing a debt in carbon credits. *Timeline is hypothetical and only reflects the duration of the forest transition within each country

A statement of the large range of carbon credits attributed in function of the baseline rule chosen was already provided by Griscom et al. [7], showing differences of two orders of magnitude in credited emissions for a same amount of effective emissions reduction, thus confirming the risk of inefficiency of REDD+ mechanism. Facing those uncertainties, Griscom et al. [7] defended the use of a strictly historical reference scenario which was an accurate predictor of deforestation in the dataset used based on FAO data. This historical baseline has been the most frequently chosen reference scenario, for its simplicity and transparency [9]. However, historical scenarios have faced a fundamental shortcoming under the perspective of forest transition, i.e. the idea that countries having experienced high rates of deforestation will decrease their deforestation rates while developing [10], making thus past deforestation a poor predictor of future deforestation (Fig. 1). By nature, under the assumptions of the forest transition theory, historical baseline does not provide incentives for High Forest Low Deforestation (HFLD) countries, which would necessarily increase their level of deforestation in the future. On the contrary, it is much more interesting for countries having experienced high past deforestation rates, by allowing them to establish high future reference levels. Although the forest transition theory has been criticized for its lack of generalizability [11], its conceptual framework is often tangible in debates related to REDD+ baselines definition [12].

HFLD countries contain a significant carbon stock, corresponding to 10.5% of tropical forest carbon [7]. They represent the largest remaining tracts of intact and unfragmented tropical forests [13]. These countries are a challenging object for the credibility and equity of REDD+ mechanism. Indeed, they might contribute to a significant increase in future deforestation if the forest transition theory is true. Furthermore, reference scenarios influenced by vested interests might exaggerate the baselines considered. However, besides this importance, they have attracted low interest of researchers within the scope of REDD+ . It is thus important to fill the scientific gap concerning these countries, especially since the publication of new deforestation maps at high temporal and spatial resolutions by Hansen et al. [14] now allows to better estimate and map the small scale deforestation observed in these areas. Based on this new dataset, a recent study focusing on the Guiana Shield (which is a highly forested area suffering low historical deforestation) showed that, contrary to the statement of Griscom et al. [7], deforestation was not decreasing but rapidly increasing under the pressure of gold-mining [15]. Moreover, such increase was shown to be very heterogeneous at the national scale, following the heterogeneity in the local political response to illegal gold-mining: talking of HFLD countries is convenient at the global scale but should not hide the diversity of local contexts.

The Guiana Shield, along with Belize, Gabon and Peru, is part of what it commonly considered as HFLD countries [7]. Guyana, Suriname, French Guiana and the Brazilian State of Amapá cover an extent of close to 600,000 sq. km of tropical rainforests and are the territories with highest forested area per capita in the world [16]. Moreover, they are a symbol of the fairness concern related with REDD+ : HFLD countries didn’t benefit from the potential economic prosperity which would have been brought by deforestation, and would need financial compensation to develop [17]. In order to allow HFLD countries to benefit from REDD+ , alternative scenarios to the historical baseline were proposed. An extreme example is the ‘Economically Rationale Baseline’ proposed by the consulting firm McKinsey & Company and published with Guyana as a case study [17], consisting of a sort of massive adjustment factor compared to the strictly historical scenario. Such a baseline, built upon McKinsey’s carbon mitigation cost-curve presenting reductions of emissions derived from slash-and-burn agriculture as a low-cost mitigation option, assumes the deforestation of all available forest areas outside protected and Amerindian areas. This legitimates the fear of ‘hot-air’ and might be considered as environmental blackmailing [18] representing a threat to the functioning of REDD+ [19].

Besides their common status of HFLD countries, these entities greatly differ in terms of legal status and structure of their economy that makes of them a study area of particular interest for the analysis of REDD+ . While Guyana and Suriname are independent countries, French Guiana and Amapá are part of a larger nation which might cause discrepancies between local and national political and administrative contexts. They also differ in terms of the structure of their economy, which is directly related to the local drivers of deforestation. In Guyana, gold-mining is considered by far as the main driver of deforestation, causing 90% of total deforestation following official figures [20], and is a major contributor to the national Gross Domestic Product (GDP), with a share of 15.5% in 2011 [21]. In Suriname, this situation is very similar: in 2010 around 10% of the national GDP was attributed to gold-mining, a value which could have increased a lot given the subsequent explosion of gold-prices in 2011 and 2012 [22]. It is also estimated that gold-mining monopolizes more than a third of the total work force in the country [23]. In French Guiana, the dependence upon natural resources is lower, as local consumption remains largely dependent upon imports from Metropolitan France [24]. Services, including transportation, provide three-fourths of the added value to the economy [25]. In Amapá also, the service sector contributes to 80% of the GDP of the State [26]. In both of these entities, repressive policies against illegal gold-mining [27, 28] might have contributed to its lower impact on the economies and on deforestation [15]. Regarding REDD+ implementation, countries’ situations are diverse besides their biophysical similarities. As previously mentioned, Guyana has been pioneer with the signing of the agreement with Norway. Suriname is now officially in an ‘implementation step’ but information about its national strategy is hardly available. Both in Amapá and French Guiana, local authorities want to propose local REDD+ projects, but they depend on the national environmental policy of Brazil and of France (which itself is not eligible for REDD+ funding by being an Annex I country).

The objective of the present study, the first one to exclusively focus on HFLD countries, was thus to assess the heterogeneity of predicted future deforestation in the Guiana Shield using different possible methodologies of reference scenarios formulation. The range of scenarios considered encompasses the different normative scenarios (i.e. describing a pre-specified future, such as defined by the IPCC [29]) used by Griscom et al. [7], where future deforestation is estimated based on past deforestation trends or basic assumptions, but also includes proposals made within the Guyana/Norway agreement. Moreover, as it is crucial that deforestation models explicitly focus on the socio-economic and political drivers of deforestation [30], we also included future deforestation scenarios where deforestation is estimated based on assumptions concerning local deforestation drivers.

The great heterogeneity of the normative scenarios indicates that any baseline could be chosen and creates a serious risk of hot air (false emissions reductions). Although an ‘Economically rationale baseline’ such as defined by McKinsey [17] is an extreme case, more balanced scenarios still predict major increases in deforestation. This could promote deforestation leakages to HFLD countries by attracting agricultural activities previously occurring within high deforestation countries, while still receiving money from carbon credits. Identifying present drivers of deforestation might create more conservative scenarios; however the contribution of gold prices, which are by nature highly volatile, makes the formulation of a credible scenario a difficult issue. In summary, predicted emissions baselines hide the main important issue which is how to support a more sustainable endogenous development for those countries. Relying on implausible baselines undermines the credibility of efforts to decrease deforestation while enhancing socio-economic development, which is necessary to eradicate poverty and in parallel to provide a long term protection to the environment.

## Methods

### Deforestation dataset

Data on deforestation in this area are based on yearly deforestation maps during 2001–2014 provided by Hansen et al. [14]. As such, we exclusively focus on deforestation and do not consider forest degradation, although the detection of deforested pixels might be produced by a continuous degradation process. These maps were re-projected to EPSG:3857 and re-sampled at a pixel resolution of 30 meters. A crown cover threshold of 75% was applied to the forest cover map of year 2000, using a strict forest definition consistent with the density of the rainforest of the Guiana Shield. We observed that below 80%, total forested area measured is not significantly changed compared to lower crown cover thresholds often used (results not shown). A majority filter was then applied to remove isolated deforested pixels most likely caused by misclassification of satellite images [31] or isolated blowdowns [32]. Large scale deforestation occurring within coastal swampy areas and mangroves was also removed, as we assumed that land use change observed in these areas was natural and not anthropogenic.

### Scenarization

Based on observed deforestation in the region over 2001–2014, we formulated different scenarios of future deforestation until 2050, described in Table 1 and more detailed in Additional file 1. We distinguished between normative and socio-economic scenarios. Normative scenarios are defined a priori, based on certain assumptions applied to past deforestation rates. These normative scenarios were built following different crediting baselines proposals. As such, these proposals initially don’t aim at predicting deforestation itself, but at being confronted with observed deforestation during the course of a REDD+ project to estimate carbon credits paid to a country. In the present study, we estimated the amount of deforestation envisioned within each of these normative scenarios to show the range of possible deforestation trends they consider, with no objective of being realistic or for predictive purposes. On the contrary to the normative scenarios, future deforestation under the different socio-economic scenarios is modeled based on the relationship between relevant socio-economic variables (population increase and gold price [30]) and deforestation.

**Table 1:** Description of the future deforestation scenarios formulated within this study

Name	Assumption	Model	References
Normative models			
Historical average (HA)	Past observed yearly deforestation over 2001–2014 continues until 2050	$CDef_{t, c}^{HA} = logN\left( {Def_{c}^{HA} + \log \left( {t + 1} \right); \sigma } \right)$ with *CDef*_*t*, *c*_^*HA*^ the cumulated deforestation over *t0*−*t*, for country *c* under the *HA* scenario, and *Def*_*c*_^*HA*^ and σ are the model parameters. *Def*_*c*_^*HA*^ was estimated as the yearly average observed deforestation in country *c* during 2001–2014	
Economically rationale baseline (ERB)	All forested areas excepted integrally protected areas and indigenous territories are deforested by 2050	$CDef_{t,c}^{ERB} = \log N\left( {Def_{c}^{ERB} + \log \left( {t + 1} \right);\sigma } \right)$ with *CDef*_*t*, *c*_^*ERB*^ the cumulated deforestation over t0−t, for country *c*, under the *ERB* scenario. *Def*_*c*_^*ERB*^ corresponds to the log of the total area assumed to be deforested in country *c* divided by 35 (so that all available lands would be deforested between 2015 and 2050)	[17]
Joint Research Center Proposal (JRC)	Countries adjust their level of deforestation to half of the global average, assumed to be linearly decreasing and reach 0 in 2050 (JRC2050) or 2100 (JRC2100)	$CDef_{t,c}^{JRC} = logN(\mathop \sum \limits_{2015}^{t} Def_{t,c}^{JRC} , \sigma )$ with $Def_{t,c}^{JRC} = (\frac{1}{2}WDR_{0} - \alpha t) \times FC_{t,c}$ *WDR*_0_ is the world annual initial deforestation rate. Within the present study, we chose its value according to estimates used within the Guyana-Norway agreement, corresponding to deforestation rates in developing countries only, and giving a value of 0.52% [33]. α = 0.0029 is the coefficient associated with the linear decrease in world deforestation rates (reaching zero deforestation in 2050 or 2100). *FC*_*t*,*c*_ corresponds to the forest cover of country *c* at time *t* in hectares	[34]
Combined Incentives (CI)	Scenarios proposed within the Guyana/Norway agreement. The Business-As-Usual scenario (CI-BAU) assumes an annual deforestation rate equal to half of the deforestation rate of developing countries, or 0.275%. The most ambitious scenario, where full payments (FPS-CI for Full Payment Scenario) would be granted to Guyana assumes a yearly deforestation rate of 0.056%. Payments were assumed to decrease if deforestation increased above the FPS-CI, reaching value 0 for an annual deforestation rate above 0.1% (NPS-CI for No Payment Scenario)	${\text{CDef}}_{{{\text{t,c}}}}^{\text{CI}} = {{\text{logN}}(\sum \limits_{2015}^{\text{t}}} {\text{Def}}_{{{\text{t,c}},}}^{\text{CI}} ;\sigma )$ with ${\text{Def}}_{{{\text{t}},{\text{c}}}}^{\text{CI}} = {\text{CI}}^{\text{S}} \times {\text{FC}}_{{{\text{t}} - 1,{\text{c}}}}$ CI^S^ is the rate of deforestation assumed in each scenario s (among BAU, FPS or NPS). FC_t-1,c_ is the forest cover in country c at time t−1 in hectares	[33]
Socio-economic models			
Gold-mining model (GM)	Yearly deforestation was explicitly modelled using population increase and gold prices as explanatory variables. Only one hypothesis was made regarding population increase within each country, while two scenarios were formulated with a low (GM-low) and high (GM-high) gold price, corresponding to the yearly average price over 2001–2014 and the double of the maximum price over the same period respectively (3077 USD/ounce)	${\text{Def}}_{{{\text{t}},{\text{c}}}} = {\text{Def}}_{{{\text{t}},{\text{c}}}}^{\text{GM}} + {\text{def}}_{{{\text{t}},{\text{c}}}}^{\text{Dem}}$ with ${\text{Def}}_{{{\text{t}},{\text{c}}}}^{\text{GM}} = {\text{logN}}(\uptheta_{{0,{\text{c}}}}^{\text{GM}} +\uptheta_{\text{c}}^{\text{V}} \times \log \left( {{\text{GoldPrice}}_{\text{t}} } \right), \sigma^{\text{GM}} )$ and ${\text{Def}}_{{{\text{t}},{\text{c}}}}^{\text{Dem}} = {\text{logN}}(\uptheta_{0}^{\text{Dem}} +\uptheta_{1}^{\text{Dem}} \times \log \left( {{\text{PopCh}}_{\text{c}} } \right);\sigma^{\text{Dem}} )$	[15, 30]

All models calibrated are log normal, as we assumed a multiplicative error term with increasing predicted deforestation. For all normative models, we estimated cumulative deforestation (CDef) by country at time t over the period of interest (2015–2050). Historical Average (HA) was the first normative model we calibrated on historical data, assuming unchanged deforestation rate in time. This model allowed us to estimate the parameter σ, corresponding to the model’s error term, which was then injected in all following normative models to take into account the uncertainty associated with predicted deforestation. Concerning the socio-economic models, yearly future deforestation was estimated by the addition of two components (Def^GM^ and Def^Dem^). Def^GM^ estimates deforestation due to gold-mining in country *c* at time *t* in function of future estimated gold prices (with high and low hypotheses), which is known to be a very good predictor of deforestation due to gold-mining [15]. Def^Dem^ estimates deforestation not due to gold-mining, assuming that remaining deforestation is a function of the demographic increase.

### Future deforestation maps

Future deforestation maps were computed by coupling a deforestation location model, predicting where deforestation is more likely to occur, and an intensity model predicting the amount of deforestation. The deforestation location model provided a spatial deforestation risk map based on Random Forest algorithm [35], known for its good predictive accuracy and robustness to noise [36], and ability to take into account complex and nonlinear relationship between deforestation and associated explanatory variables [37]. Explanatory variables included in this model were all geographical variables listed in Table 2. Greenstone areas are more favorable to the presence of gold, we thus expect a decreasing probability of deforestation due to gold-mining further from Greenstones. Alluvial gold-mining is the dominant exploitation method in the region: deforestation due to gold-mining is likely to concentrate along small streams of low Strahler order. The predicted intensity of deforestation, i.e. the number of pixels to sample from the deforestation risk map to obtain the final predicted deforestation map, was derived from the different above-mentioned deforestation scenarios. Maps were finally computed for those nine different scenarios. For more details on the methodology applied here to create future deforestation maps, please refer to Dezécache et al. [30].

**Table 2:** List of geographical explanatory variables included in the deforestation location models

Variable name	Resolution (m)	Approx. range	Sources
Protected areas	30	Binary	*See legend
Distance to nearest road	150	0–170 km	**See legend
Distance to nearest Greenstone area	150	0–65 km	**See legend
Distance to nearest stream following Strahler classication:			[38–40]
Order 1–3 (small)	150	0–2 km	
Order 4–6 (intermediate)	150	0–15 km	
Order 7+ (large)	105	0–120 km	

* Shapefiles of protected areas and road network were provided respectively by Forest offices in Guyana (GFC), Suriname (SBB), French Guiana (ONF) and Amapa (IEF). ** The shapefiles for Greenstone areas were manually digitized following the geological map produced by the Guyana Geology and Mines Commission (http://www.ggmc.gov.gy/Documents/PDF/GeoServices/guy_geol.pdf) in Guyana; obtained from the Surinamese forest office (SBB) in Suriname; obtained from French Geological Survey (BRGM) in French Guiana; and provided by the Scientific and Technological Research Institute (IEPA) in Amapá

### From deforestation scenarios to carbon crediting

In order to estimate the amount of carbon credits which could be paid to a country following different reference scenarios assumed, we estimated the difference between mean predicted deforestation over 2015–2050 from each scenario and the historical scenario. We assumed that the pathway effectively followed by each country corresponded to the historical baseline, as we were interested in the amount of credited carbon in the case where countries manage to stabilize their deforestation rates to low historical values.

Estimated avoided deforestation over 2015–2050 allowed us to calculate avoided carbon emissions by using a carbon density of aboveground biomass of 132.5 tC/ha, based on an average 265 t/ha of dry aboveground biomass over the area and a 50% coefficient to convert this dry biomass to carbon content, as reported in Molto [41]. A coefficient of 44/12^1^ was applied to convert these tons of carbon into tons of CO_2_. Two carbon prices were used: a low price of 5 USD/t close to current carbon price within the EU Emissions Trade System and a high price of 30 USD/t proposed by France as a carbon price floor in Europe.^2^ Potential incomes due to the sale of carbon credits obtained from avoided deforestation, including their uncertainty for the different scenarios and possible carbon prices, were finally expressed in  % of average national yearly GDP over 2001–2014.

## Results

### Variability of predicted deforestation intensity under each baseline

**Fig. 2:**
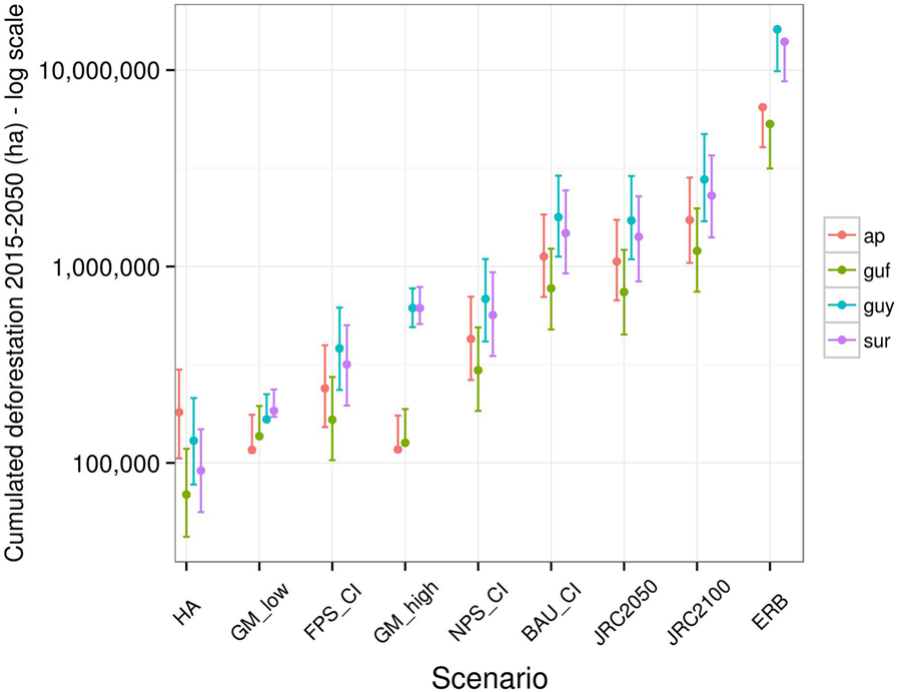
Cumulated predicted deforestation over 2015–2050 per country (‘ap’, ‘guf’, ‘guy’ and ‘sur’ stand for Amapá, French Guiana, Guyana and Suriname respectively) and per scenario (log y-scale). Dots are mean predicted deforestation and are associated with a 95% confidence interval. ‘HA’ stands for ‘Historical average’. ‘GM_low’ and ‘GM_high’ stand for the gold-mining models with a low or high assumed future gold price. ‘ERB’ stands for ‘Economically Rational Baseline’. ‘CI’ stands for Combined Incentives models (with three sub-models FPS, NPS and BAU described in Table 1). ‘JRC’ stands for ‘Joint Research Center’ with two associated sub-models for 2050 and 2100

**Fig. 3:**
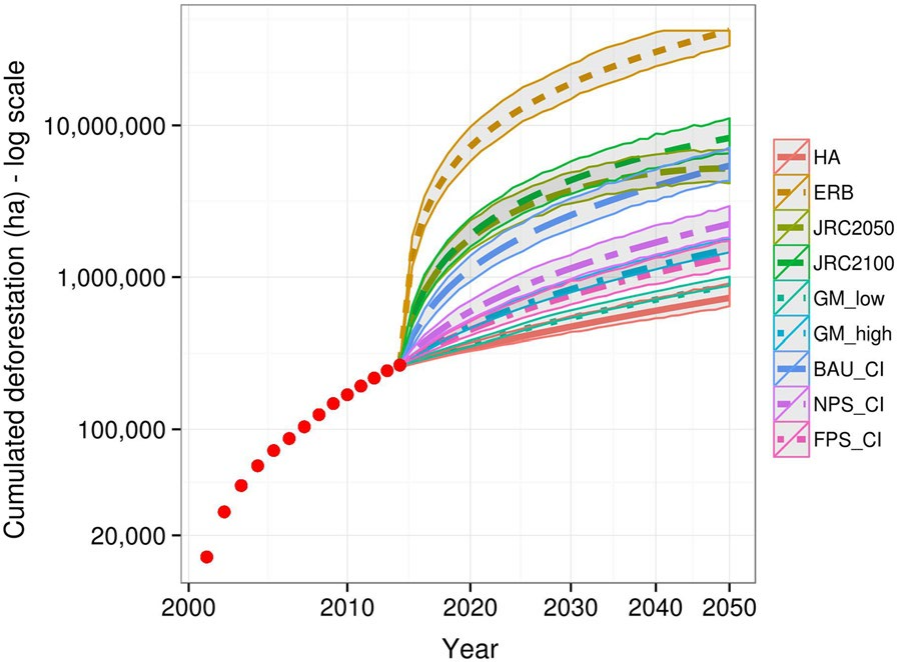
Cumulative predicted deforestation at the regional scale (2015–2050) following each reference scenario (log y and x scales). Red dots indicate observed yearly deforestation over 2001–2014. Mean predicted future deforestation is displayed with an envelope corresponding to the 95% confidence interval of each model. ‘HA’ stands for ‘Historical average’. ‘GM_low’ and ‘GM_high’ stand for the gold-mining models with a low or high assumed future gold price. ‘ERB’ stands for ‘Economically Rational Baseline’. ‘CI’ stands for Combined Incentives models (with three sub-models FPS, NPS and BAU described in Table 1). ‘JRC’ stands for ‘Joint Research Center’ with two associated sub-models for 2050 and 2100

Predicted deforestation is extremely variable following the scenario applied, with a range of two orders of magnitude between the lowest normative scenarios and the Economically rationale baseline (ERB, see definition in Table 1) whether at national (Fig. 2) or regional scales (Fig. 3). Even excluding the extreme case of the ERB, we still observe a difference of more than one order of magnitude between lowest and highest future deforestation scenarios. Both socio-economic scenarios are among the low deforestation scenarios, but their predictions greatly differ between the low and high gold price hypotheses in Guyana and Suriname where deforestation is largely determined by gold-mining activity.

### Future deforestation maps

**Fig. 4:**
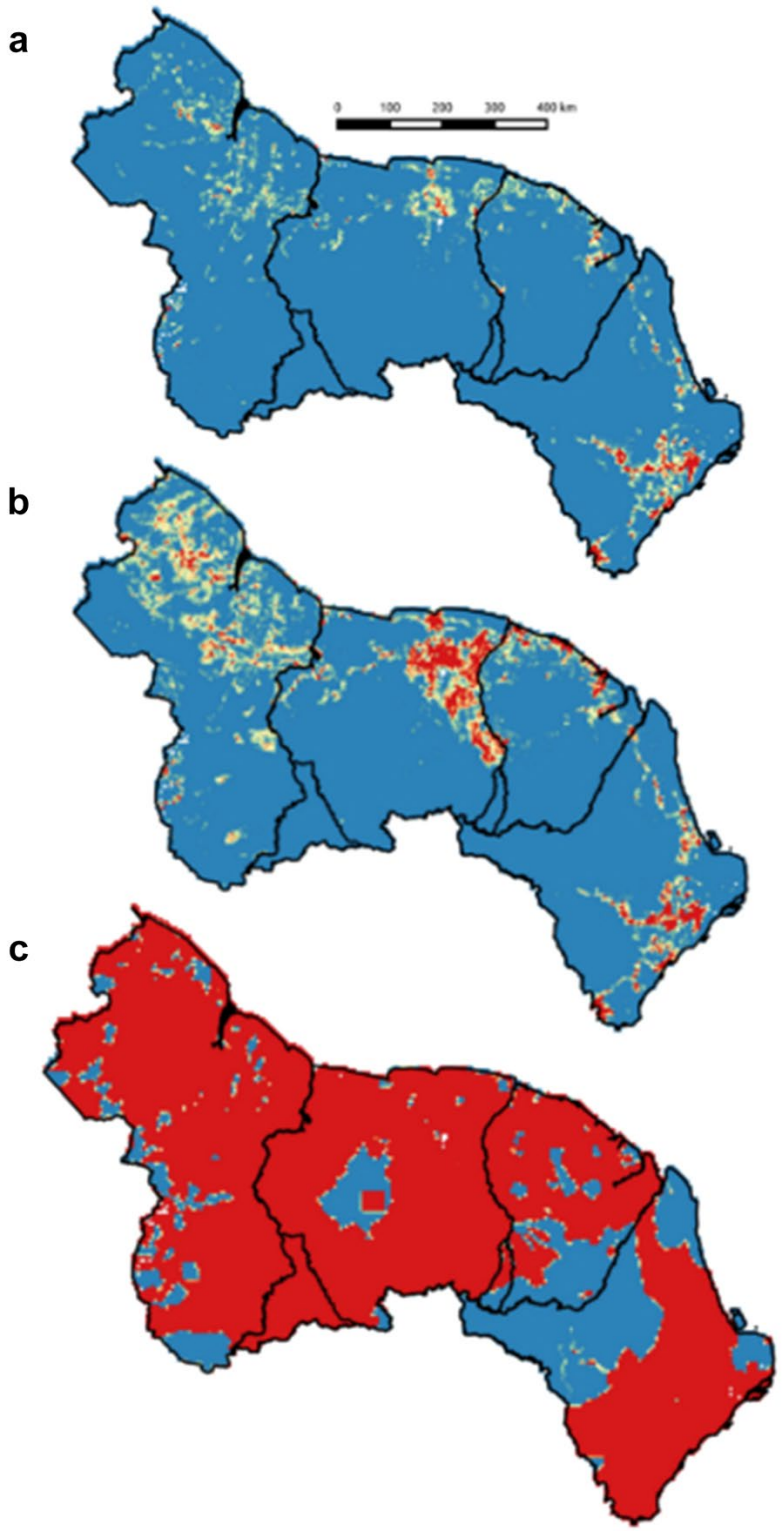
Maps of future predicted deforestation under historical scenario (**a**), high gold price scenario (**b**) and ‘Economically Rational Baseline’ (**c**) in Guyana, Suriname, French Guiana and Amapá (left to right). Red pixels correspond to areas of high deforestation (> 30% of 2014 forest cover deforested over 2015–2050). Border conflicts between Guyana and Suriname, and Suriname and French Guiana cause an overlap in the southern parts of these countries

Directly related with those huge differences in terms of predicted deforestation, future deforestation maps (maps for all scenarios are displayed in SM3) are very variable, especially comparing historical or high gold price scenarios (Fig. 4a, b) with the extremely high ERB scenario (Fig. 4c) where, by definition, only integrally protected areas and Amerindian areas remain forested. Besides changes in predicted deforestation intensity, a visual comparison of historical (Fig. 4a) and high gold price (Fig. 4b) baselines also indicates major shifts in the location of deforestation hotspots. Indeed, compared to the historical scenario, large amounts of deforestation appear in Suriname and Guyana in areas corresponding to Greenstone areas where gold-mining activities are concentrated.

**Fig. 5:**
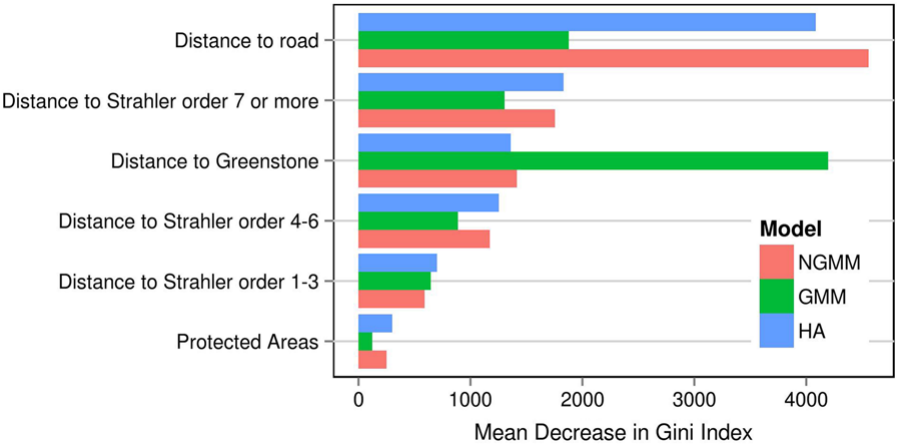
Ranking following Mean Decrease in Gini index (MDG) of spatial variables included within the three deforestation location models. NGMM, GMM, HA stand for no gold-mining model, gold-mining model and historical scenario respectively. Mean Decrease in Gini index (MDG) is used to rank variables by importance. When random forest algorithm is used to classify a sample of pixels within two groups (deforested, not deforested), a very important variable will increase a lot the “purity” within each group and will be associated with a large MDG. On the contrary, a variable of low importance will not contribute to increasing intra-group purity and will have a lower MDG

Such shift in deforestation location is also supported by the ranking by index of importance of variables included within the different Random Forest models (Fig. 5). In the deforestation location component of the historical scenario, the most important variable is distance to nearest road. Under the high gold price scenario, two deforestation location models were calibrated, one for deforestation due to gold-mining and another one for deforestation not due to gold-mining. Variables ranking within the model for not gold-mining areas is similar to the ranking of the historical model. However, in gold-mining areas, distance to Greenstone appears as the most important variable, which greatly shapes the deforestation map as, with extremely high gold prices, gold-mining largely increases its contribution to deforestation in Guyana and Suriname compared to the historical scenario.

### The contribution of credited carbon to national GDP

**Fig. 6:**
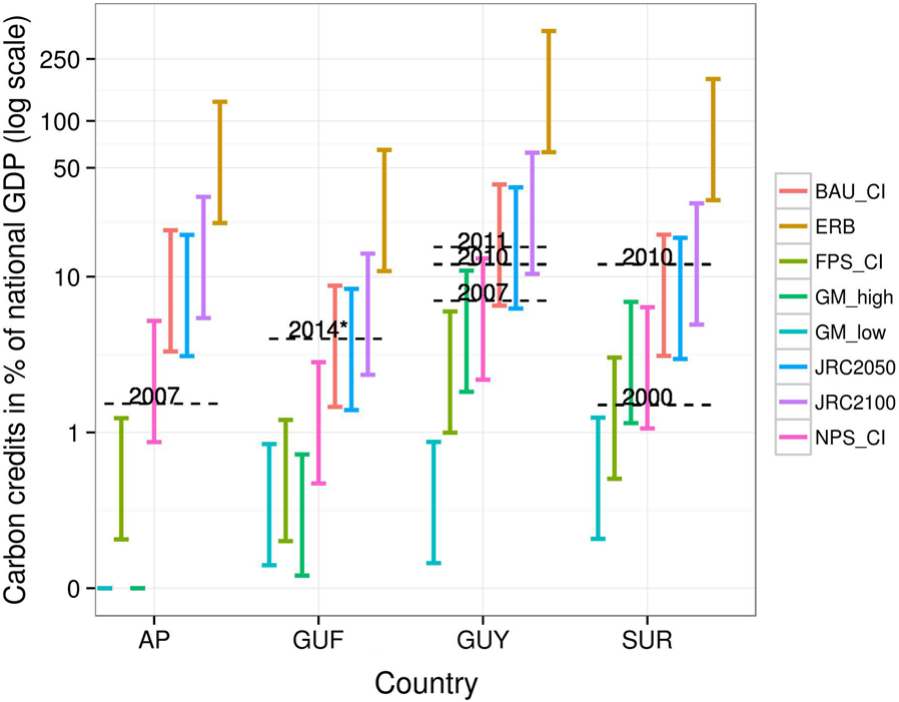
Quantity of allowable cumulated carbon credits up to 2050, following each alternative deforestation baseline, and expressed as the share of mean yearly national GDP over 2001–2014 (log scale). For each scenario, the range indicates the share of GDP for carbon prices ranging between 5 and 30 USD/tCO_2_e. ‘AP’, ‘GUF’, ‘GUY’ and ‘SUR’ stand for Amapá, French Guiana, Guyana and Suriname respectively. We assume no debt in case of scenario followed lower than historical average, which explains values 0 attributed to GM-low and GM-high scenarios in Amapá. As a comparison, the contribution of gold-mining to national GDP [21, 23, 26] was added to this figure in the form of black dotted segments, with corresponding year indicated above each segment. This data was uncertain for French Guiana as taken from an interview (https://reporterre.net/La-foret-guyanaise-menacee-par-les-mines-d-or) and not available in official databases, and was denoted with an asterisk. ‘HA’ stands for ‘Historical average’. ‘GM_low’ and ‘GM_high’ stand for the gold-mining models with a low or high assumed future gold price. ‘ERB’ stands for ‘Economically Rational Baseline’. ‘CI’ stands for Combined Incentives models (with three sub-models FPS, NPS and BAU described in Table 1). ‘JRC’ stands for ‘Joint Research Center’ with two associated sub-models for 2050 and 2100

The very diverse contribution of each hypothetical REDD+ scenario to each country’s GDP reflects the variability of predicted deforestation (Fig. 6). The assumed range of carbon prices (5 to 30 USD/tCO_2_e) also strongly affect this estimated percentage. A comparison with the contribution of gold-mining to national GDP indicates that only extremely high reference scenarios such as ERB, JRC2050 and 2100, and BAU-CI might compensate the contribution of gold-mining, especially for more recent years where incomes provided by gold-mining increased a lot with the explosion of gold prices.

## Discussion

### The credibility of future deforestation scenarios

#### The footprint of the forest transition theory in REDD**+** debate on the formulation of deforestation reference levels

Angelsen and Rudel [12] suggest that there should be no ‘one-size-fits all portfolio of REDD+ policies’ and that the forest transition theory is a useful tool for adapting REDD+ to the differing national contexts. As such, while in countries at an early stage in the forest transition (like HFLD countries, assuming that their high forest covers prove that they haven’t passed the forest transition yet) REDD+ should focus on preserving carbon stored within old-growth forest, in countries experiencing high rates of current deforestation, such as Indonesia, the emergency would be to slow down deforestation rates. Finally, in countries with low remaining forests, incentives for forest regrowth would be the priority in terms of national carbon balance. Although such flexibility would enhance countries participation, which is necessary for REDD+ to be effective, it could seriously reduce its efficiency if the forest transition theory is not (or not anymore) a pertinent concept, by authorizing very high baselines for countries identified as HFLD.

Angelsen and Rudel [12] mention different socio-economic and political determinant of forest transition, but in our opinion the debate remains too closely associated to the questions of forest cover and deforestation only, such as suggested by the mentions to HFLD, HFHD, LFHD and LFLD countries which might be perceived as successive necessary steps in the environmental History of a country. Such a denomination should not be used as a too simplistic trademark unable to appropriately characterize the underlying socio-economic and political processes occurring within the different countries. As previously mentioned, the service sector contributes to more than 3/4 of the GDP of Amapá and French Guiana. Additionally, the urbanization rate has been increasing a lot in Suriname, from 49% in 1975 to 74% in 2005 [42]. Similarly in French Guiana, the two cities of Cayenne and Kourou account for 73% of the population [42]. Such a concentration in main cities is also obvious in Amapá, where 75% of the population are concentrated in the capital district of Macapa, making Amapá the most urban State of Brazil [43]. Only in Guyana the rural population represents a very significant share of the population, with only 27% of the population being urban in 2005 [44]. Such a low percentage of rural inhabitants in the region, itself inhabited by a very low population, questions the credibility of the development of large scale agriculture in the absence of available work force, although recruitment of foreign labor cannot be discarded.

#### Confronting inflated baselines to local evidences from deforestation data in the Guiana Shield

Besides the general socio-economic background in the region, our socio-economic scenarios suggest that current drivers of deforestation in the countries involved, mostly gold-mining, urban and agricultural expansion, are unlikely to provoke huge increase in deforestation such as observed in the extremely high normative scenarios, even in a context of very high gold prices. Such a statement is of course questionable in the framework of the forest transition theory, because in these socio-economic modelling frameworks we didn’t assume the possibility of great shifts in development trends.

Export agriculture is likely to be the only driver which could cause such high deforestation as predicted from high normative scenarios, in particular oil palm plantations which are included as a major component of potential deforestation in the report by McKinsey [17] over Guyana.

Oil palm plantations exist in Guyana and Suriname but at a very small scale. In Suriname, more than 5000 ha of oil palm plantations were reported in 1990, a figure which dropped to 20 ha in 2000, due to the political instability and a bud rot disease present in Central and South America [45]. Some argued that oil palm might rise again in Suriname, after an agreement was signed in 2004 with a Chinese company for exploiting 40,000 ha.^3^ Although the project was delayed for more than 10 years, the government of Suriname declared that a new agreement would be signed soon [46]. However, given the extremely low soil fertility [16, 47], and low level of infrastructures in the Guiana Shield, it is questionable whether such a large scale expansion of oil palm in the region is a credible scenario, in particular within a context of development of zero deforestation objective in the oil palm industry.^4^ Developing countries might fail to implement the necessary programs to effectively limit their deforestation rates [18], but inversely it is questionable whether they can effectively provoke such increase in deforestation as predicted based on the high normative scenarios, because such increase would not only be a question of political will but would also depend on the existence of potential economic investors.

### The harmful consequences of disproportionate deforestation scenarios

#### Corrupting REDD**+** additionality principle

The extreme variability of deforestation baselines under different procedures of reference scenarios formulation, such a previously noted by Griscom et al. [7] makes the choice of a baseline non trivial compared to simple historical average, which provides strong incentives for major deforesting countries only. Given the range of possible baselines, it is impossible to clearly state which would be the most appropriate scenario in the absence of any consensual choice criteria. This is even more critical in the case of HFLD countries, which are not in a good situation for bargaining compared to major deforesting countries. Indeed, while they need to demonstrate that their future rates of deforestation will increase compared to historical rates, in order to benefit from higher financial transfers from eventual REDD+ projects, a risk of baseline inflation emerges [8].

Such risk of baseline inflation greatly threatens the additionality principle which is one of the major pillars of REDD+ mechanism. This principle is also questioned by the natural uncertainty of the modelled deforestation process, such as evidenced by the comparison of our socio-economic scenarios. While deforestation remains almost unchanged in Amapá and French Guiana in the low and high gold prices scenarios, as both countries have been fighting against illegal gold-mining [27], a major increase in deforestation is predicted in Suriname and Guyana in the case of high gold price (multiplication by a 2.9 factor compared to the low price scenario). While there could be a tendency to assume that low deforestation within countries of the Guiana Shield persists due to a strong political will (this is perceptible in press articles indicating that Guyana ‘kept’ deforestation low [48]), it is critical to pay attention to biases caused by changing commodities prices which can alter the perception of local political efforts, as for example if deforestation remains at low values not due to the implementation of stronger policies but because of low gold prices.

Finally, if a development of agribusiness cannot be excluded a priori in the Guiana Shield, including this option within incredibly high reference levels might create a risk of global deforestation leakages, which would also affect REDD+ additionality at the global scale. Deforestation due to oil palm plantations could leak from present high deforestation countries to HFLD countries which could continue to receive large amount of carbon credits while increasing a lot their deforestation rates. This possibility would seriously undermine the efficiency of REDD+ mechanism.

#### Will REDD**+** funds be enough to effectively limit deforestation?

The success of emissions-based approaches such as REDD+ is based on the hypothesis that reducing deforestation and forest degradation is possible at low cost [3, 4, 17]. However, opportunity costs are really underestimated, because they cannot include non-markets activities such as subsistence shifting cultivation. More importantly, they neglect implementation, transaction and institutional costs which might represent the hidden part of the iceberg [19]. As an example, a case study from Brazil estimated that direct payments to families and support to communities accounted for 55% of the total cost of the project, the remaining being associated with administrative or enforcement costs [19].

This point will be critical in the case of the Guiana Shield, where a large share of deforestation derives from artisanal small-scale gold-mining [49]. This activity might be tolerated de facto in most cases but could instantaneously convert to an officially illegal activity in case of higher level of law enforcement. In case of illegal activities, Gregersen et al. [50] suggest that the opportunity cost is inappropriate, and that the cost of a deforestation reduction project is simply the cost of law enforcement. French Guiana has been involved in a repressive policy against illegal gold-mining for more than 10 years [27], aiming at decreasing the environmental impacts of such activity. The costs of such policy are confidential but are likely to be huge as it includes satellite and helicopter observation of mining sites over a very large area covering thousands of sq. km, terrestrial interventions to destroy illegal mining sites and in some case the deportation of illegal Brazilian gold-miners who represents a great majority of the gold-mining labor force in the region [51]. This point is likely to be underestimated when blind historical models are computed in the form of future deforestation maps without characterizing the underlying socio-economic drivers of deforestation [30]. In our study area, these models represented by our historical average model, are mostly influenced by the tendency of roads to focalize deforestation hotspots in accessible areas, forgetting the existence of gold-mining in very remote areas difficult to monitor and control. Moreover, beside monitoring and repression costs, ensuring a permanence of avoided deforestation would eventually also require costs associated with finding alternative jobs for people previously involved in illegal activities [52].

Finally, beyond the problem of estimating the total costs of avoided deforestation, which is likely to be large, it is necessary to question the ability and the willingness of donors to compensate for avoided deforestation estimated based on a chosen reference scenario. As previously mentioned, the example of the Government of Norway, which only paid a fraction of the opportunity cost of not deforesting Guyana estimated by McKinsey & Company, might reveal that funds available are limited. While McKinsey’s report estimated that an annuity comprised between 430 million and 2.3 billion USD might be enough to compensate for the opportunity costs of not deforesting Guyana [17], Norway finally agreed to provide only up to 50 million USD per year on average, over a project of 5 years. If several donors must contribute together to reach the required funding level, the convergence of their requirements might also be difficult to obtain.

## Conclusions

Focusing on the underlying processes leading to deforestation, rather than simply predicting deforestation patterns based on historical models, might contribute to a better understanding of what would be needed to make REDD+ effective [53]. As previously mentioned, opportunity costs might greatly underestimate real costs of avoided deforestation, and we can wonder whether funds provided from REDD+ carbon credits will be enough to develop such policies, unless extremely high reference scenarios are formulated in order to attract huge amounts of financial incentives. Even though substantial financial transfer are made however, we may wonder if countries can succeed in implementing the necessary policies to decrease their rates of deforestation, especially in case of poor or ‘failed States’ [18]. Indeed, moving from a simple conceptual framework, where land-users were paid to limit the impact of their activities on neighboring forest, to a much more complex situations were perverse incentives and corruption are perceived as leading forces of deforestation [5, 18] points out the importance of the political dimension of deforestation.

Although it is tempting for HFLD countries to elaborate inflated references levels for REDD+ to make this mechanism more incentive compared to a strictly historical baseline, these countries could lose credibility and be accused of environmental blackmailing. Addressing current drivers of deforestation instead of very hypothetical ones is necessary and sufficient, with no need to exaggerate baselines, as limiting their impact will be difficult and costly. The only way to ensure permanence of REDD+ credits is by changing the development trends of forested countries and eliminating corruption and perverse incentives to deforest, which cannot be expressed in the form of speculative future deforestation scenarios. These scenarios may be useful, but they can only be part of a larger bundle of information and arguments that should encompass a variety of hypothesis underlying several plausible scenarios.

## Additional file

**Additional file 1.** Detailed explanation concerning the different future deforestation scenarios formulated within this study.

## Abbreviations

CI: Combined Incentives model
ERB: Economically Rationale Baseline
GM: gold-mining model
JRC: Joint Research Center model
REDD+: reducing emissions from deforestation and forest degradation

## Footnotes

1 https://www.epa.gov/energy/greenhouse-gases-equivalencies-calculator-calculations-and-references.

2 https://www.theguardian.com/environment/2016/may/17/france-sets-carbon-price-floor.

3 http://www.caribbeannewsnow.com/headline-Suriname-palm-oil-industry-may-rise-again-after-setbacks-32970.html.

4 https://www.theguardian.com/sustainable-business/2015/jan/26/palm-oil-companies-deliver-deforestation-promises.

## References

[1] Le Quéré C, Moriarty R, Andrew RM, Canadell JG, Sitch S, Korsbakken JI (2015). Global carbon budget 2015. Earth Syst Sci Data.

[2] Angelsen A (2008). Moving ahead with REDD. Issues, Options and Implications.

[3] Stern N (2007). The Economics of climate change.

[4] Kindermann G, Obersteiner M, Sohngen B, Sathaye J, Andrasko K, Rametsteiner E (2008). Global cost estimates of reducing carbon emissions through avoided deforestation. Proc Natl Acad Sci.

[5] van Oosterzee P, Blignaut J, Bradshaw CJA (2012). iREDD hedges against avoided deforestation’s unholy trinity of leakage, permanence and additionality. Conserv Lett..

[6] Huettner M, Leemans R, Kok K, Ebeling J. A comparison of baseline methodologies for “Reducing Emissions from Deforestation and Degradation”. Carbon Balance Manag. 2009;4(4). http://www.cbmjournal.com/content/4/1/4. 10.1186/1750-0680-4-4 PMC2717061 19594899

[7] Griscom B, Shoch D, Stanley B, Cortez R, Virgilio N (2009). Sensitivity of amounts and distribution of tropical forest carbon credits depending on baseline rules. Environ Sci Policy.

[8] Karsenty A (2008). The architecture of proposed REDD schemes after Bali: facing critical choices. Int For Rev..

[9] Pana AC, Gheyssens J. Baseline choice and performance implications for REDD. J Environ Econ Policy. 2015.

[10] Dudley RG (2010). A little REDD model to quickly compare possible baseline and policy scenarios for reducing emissions from deforestation and forest degradation. Mitig Adapt Strateg Glob Chang.

[11] Perz SG (2007). Grand theory and context-specificity in the study of forest dynamics: forest transition theory and other directions. Prof Geogr.

[12] Angelsen A, Rudel TK (2013). Designing and implementing effective REDD+ policies: a forest transition approach. Rev Environ Econ Policy.

[13] Wade TG, Riitters KH, Wickham JD, Jones KB (2003). Distribution and causes of global forest fragmentation. Conservation Ecology.

[14] Hansen MC, Potapov PV, Moore R, Hancher M, Turubanova SA, Tyukavina A (2013). High-resolution global maps of 21st-century forest cover change. Science (80- ).

[15] Dezécache C, Faure E, Gond V, Salles J-M, Vieilledent G, Hérault B (2017). Gold-rush in a forested El Dorado: deforestation leakages and the need for regional cooperation. Environ Res Lett..

[16] Hammond DS (2005). Tropical forests of the Guiana Shield : ancient forests in a modern world.

[17] Office of the President, Republic of Guyana. Saving the world's forest today: creating incentives to avoid deforestation. December 2008.

[18] Karsenty A, Ongolo S (2012). Can, “fragile states” decide to reduce their deforestation? The inappropriate use of the theory of incentives with respect to the REDD mechanism. For Policy Econ.

[19] Dyer N, Counsell S. Briefing McREDD : How McKinsey “cost-curves” are distorting REDD. Climate and Forests Policy Brief. London; 2010.

[20] Guyana Forestry Commission, Indufor. Guyana REDD + Monitoring Reporting & Verification System (MRVS). Year 3 Interim Measures Report. Helsinki; 2013.

[21] Singh D, Bernard C, Rampersaud P, Laing T, Balraj D, Priester M, et al. Guyana’s Extractive Industry Sector (EIS). A Synopsis of Issues and Recommendations for the mining sector as a Sustainable Element of Guyana’s Low Carbon Devlopment Strategy (LCDS). Georgetown; 2013.

[22] Alvarez-Berríos NL, Mitchell Aide T (2015). Global demand for gold is another threat for tropical forests. Environ Res Lett..

[23] Central Bank van Suriname. Leading sectors of suriname: the impact of mining, agriculture and tourism activities on the economy. 1970–2012. Paramaribo; 2014.

[24] Hecquet V, Moriame E. Guyane: un développement sous contraintes. Antianéchos de Guyane; 2008. p. 1–4.

[25] IEDOM. Rapport annuel 2014 Guyane. Paris; 2015.

[26] de Oliveira MJ. Mineração e desenvolvimento local : benefícios e desafios aos municípos amapaenses. Universidade Federal do Pará; 2010.

[27] de Rohan J, Dupont B, Berthou J, Antoinette J-E. La Guyane : une approche globale de la sécurité [Internet]. 2011. http://www.senat.fr/rap/r10-271/r10-2710.html. Accessed 1 June 2016.

[28] Plouvier D, Gomes L, Verweij P, Verlinden N. Living Guianas Report 2012. Paramaribo; 2012.

[29] IPCC. Climate Change 2001: impacts, adaptation, and vulnerability. 2001.

[30] Dezécache C, Salles J-M, Vieilledent G, Hérault B (2017). Moving forward socio-economically focused models of deforestation. Glob Change Biol..

[31] Mather PM. Computer processing of remotely-sensed images: an introduction [Internet]. Vol. 4. Wiley; 2004. p. 324. https://books.google.com/books?id=x0aHc4zxv74C&pgis=1. Accessed 17 May 2016.

[32] Goulamoussène Y, Bedeau C, Descroix L, Linguet L, Hérault B (2017). Environmental control of natural gap size distribution in tropical forests. Biogeosciences.

[33] LCDS Guyana. Joint concept note. Georgetown; 2011.

[34] Mollicone D, Achard F, Federici S, Eva HD, Grassi G, Belward A (2007). An incentive mechanism for reducing emissions from conversion of intact and non-intact forests. Clim Change.

[35] Breiman L (2001). Random forests. Mach Learn.

[36] Dietterich TG (2000). An experimental comparison of three methods for constructing ensembles of decision trees. Mach Learn..

[37] Evans JS, Murphy MA, Holden ZA, Cushman SA, Drew CA, Wiersma Y, Huettmann F (2011). Predictive species and habitat modeling in landscape ecology. Predictive species and habitat modeling in landscape ecology: concepts and applications.

[38] Horton RE (1945). Erosional development of streams and their drainage basins; hydrophysical approach to quantitative morphology. Geol Soc Am Bull.

[39] Strahler AN (1952). Hypsometric (area-altitude) analysis of erosional topography. Geol Soc Am Bull.

[40] USGS. Shuttle Radar Topography Mission (SRTM) 1 Arc-Second Global. 2000.

[41] Molto Q. Estimation de biomasse en forêt tropicale humide. Université des Antilles et de la Guyane; 2012.

[42] CEROM. Guyane-Suriname. Une meilleure connaissance mutuelle pour une coopération renforcée. Cayenne; 2008.

[43] Viégas H. Macrocefalia Urbana no Amapá. Realidades Urbanas. [Internet]. 2012. http://realidadeurbanas.blogspot.com/2012/06/macrocefalia-urbana-no-amapa.html. Accessed 3 Oct 2017.

[44] USAID. Urbanization in Latin America and the Caribbean: trends and challenges. 2010.

[45] De Franqueville H (2003). Oil palm bud rot in latin America. Exp Agric..

[46] Chikrie R. Suriname palm-oil industry may rise again after setbacks [Internet]. Caribbean New Now! 2016. http://www.caribbeannewsnow.com/topstory-Suriname-palm-oil-industry-may-rise-again-after-setbacks-32970.html. Accessed 3 Dec 2017.

[47] Grau O, Peñuelas J, Ferry B, Freycon V, Blanc L, Desprez M (2017). Nutrient-cycling mechanisms other than the direct absorption from soil may control forest structure and dynamics in poor Amazonian soils. Sci Rep.

[48] Norvegian Ministry of Climate and Environment. Guyana keeps deforestation low [Internet]. 2016. https://www.regjeringen.no/en/aktuelt/guyana-keeps-deforestation-low/id2480060/. Accessed 1 Oct 2018.

[49] Rahm M, Jullian B, Lauger A, de Carvalho R, Vale L, Totaram J, et al. Monitoring the impact of gold mining on the forest cover and freshwater in the Guiana Shield. 2015;(Reference year 2014):1–60.

[50] Gregersen H, El Lakany H, Karsenty A, White A. Does the opportunity cost approach indicate the real cost of REDD+ ? Rights and realities of paying for REDD+ . Washington DC; 2010.

[51] Heemskerk M, Olivieira M, Abese D, De Dehn L, Dehli C, Kasketi S, et al. Maroon perceptions of small-scale gold mining impacts, II. A survey in mining camps and affected communities in Suriname and French Guiana. Paramaribo; 2004.

[52] Hirons M (2011). Locking-in carbon, locking-out livelihoods? Artisanal mining and REDD in Sub-Saharian Africa. J Int Dev.

[53] Brown DG, Band L, Green K, Irwin E, Jain A, Pontius R (2014). Advancing land change modeling.

[54] Angelsen A. How do we set the reference levels for REDD payments ? Mov ahead with REDD. 2009;(Cdm):53–156.

