# Supplementary material for: Questioning emissions-based approaches for the definition of REDD+ deforestation baselines in high forest cover/low deforestation countries
Source: Carbon Balance Manag. 2018 Oct 30;13:21. doi: 10.1186/s13021-018-0109-1 (PMC6207608; doi:10.1186/s13021-018-0109-1)
Supplement: Supplementary file 1 — Additional file 1. Detailed explanation concerning the different future deforestation scenarios formulated within this study. [file 13021_2018_109_MOESM1_ESM.pdf]

Supplementary materials

August 23, 2018

SM1 - Detailed explanation concerning the different future deforestation scenarios formulated within this study

Normative scenarios

Seven normative scenarios were formulated:

- Historical Average (HA). Under this scenario, we assumed that past observed yearly deforestation over 2001-2014 continues until 2050. A log-normal model was calibrated, assuming a multiplicative error term with increasing predicted deforestation:

  $$CDef_{t,c}^{HA} = log\mathcal{N}(Def_c^{HA} + log(t+1), \sigma)$$

  with $CDefHA_{t,c}$ the cumulated deforestation over $t0 - t$, for country c under the HA scenario, and Def_c^{HA} and σ are the model parameters. Def_c^{HA} corresponds to the yearly average observed deforestation in country c during 2001-2014. For all following normative models, the σ inferred from the historical datasets used to set the HA scenario is used to take into account the inherent variability of the deforestation process.

- Economically Rationale Baseline (ERB). This scenario was based on the ERB formulated by McKinsey & Company (2008) in the case of Guyana. Assuming that forested areas can provide higher 'value to the nation' when converted to other land uses, this report estimates the cost of avoiding the deforestation of all forested areas in Guyana, except legally protected areas. We then applied a similar hypothesis for the four territories concerned here, assuming the deforestation of all remaining forest except integrally protected areas and Indigenous territories. The ERB model was formulated as follows:

  $$CDef_{t,c}^{ERB} = log\mathcal{N}(Def_c^{ERB} + log(t+1), \sigma)$$

  with $CDef_{t,c}^{ERB}$ is the cumulated deforestation over $t0 - t$, for country c, under the ERB scenario. Def_c^{ERB} corresponds to the log of the total area

assumed to be deforested in country c divided by 35 (so that all available lands would be deforested between 2015 and 2050).

- Joint Research Center Proposal (JRC). The JRC scenario is based on the JRC Proposal (Mollicone et al., 2007) where carbon credits are attributed to countries involved if they manage to decrease their deforestation rates under half of global deforestation rates. Under this proposal, a distinction is made between intact and non-intact forests, but due to data constraints we were not able to make such distinction. Here we assumed a linear decrease in global deforestation rates, with two alternative scenarios where zero global deforestation is reached in 2050 (JRC2050) or 2100 (JRC2100). In our JRC scenarios, we thus assume that countries adjust their level of deforestation to half of the world average. The JRC scenario was formulated as follows:

$$CDef_{t,c}^{JRC} = log\mathcal{N}(\sum_{2015}^{t} Def_{t,c}^{JRC}, \sigma)$$

with

$$Def_{t,c}^{JRC} = (\frac{1}{2}WDR_0 - \alpha t) \times FC_{t,c}$$

WDR_0 is the world annual initial deforestation rate. Within the present study, we chose its value according to estimates used within the Guyana-Norway agreement, corresponding to deforestation rates in developing countries only, and giving a value of 0.52% (LCDS Guyana, 2011). α is the coefficient associated with the linear decrease in world deforestation rates (reaching zero deforestation in 2050 or 2100). $FC_{t,c}$ corresponds to the forest cover of country c at time t in hectares.

- Combined Incentives (CI). This scenario was based on the Guyana-Norway agreement, mentioning different pathways where decreasing amounts of carbon would be credited with increasing deforestation (LCDS Guyana, 2011). This agreement made a distinction between a business-as-usual baseline (BAU) and crediting baselines. The BAU indicates how much deforestation might increase in the absence of intervention from the country involved. On the contrary, crediting baselines, set lower compared to the BAU, effectively estimates how much funds would be credited for different future deforestation rates (Angelsen, 2009). The rationale behind this choice, as stated by Guyana and Norway, is that, although deforestation in Guyana might reach the values assumed within the BAU scenario, 'neither Norway nor Guyana wishes to see such an increase in deforestation' (LCDS Guyana, 2011). The BAU scenario (hereafter called BAU-CI) was calculated here, such as done within the Guyana-Norway agreement, as the average of yearly deforestation in the area of interest for 2001-2014 and yearly deforestation rate in developing countries, assumed to be 0.52%, giving a yearly deforestation rate of 0.275%. Two different crediting baselines were considered here, one full payment scenario (FPS-CI) and

a no-payment scenario (NPS-CI), corresponding to the two extreme crediting baselines considered within the Guyana-Norway agreement, where progressively decreasing payments were proposed if effective deforestation exceeds the FPS-CI value (corresponding to a deforestation rates of 0.056% annually), reaching value 0 for deforestation equal or higher than the threshold corresponding to the NPS_CI scenario (0.1% annually). This CI scenario was formulated as follows:

$$CDef_{t,c}^{CI} = log\mathcal{N}(\sum_{2015}^{t} Def_{t,c}^{CI}, \sigma)$$

with

$$Def_{t,c}^{CI} = CI^S \times FC_{t-1,c}$$

CI^s is the rate of deforestation assumed in each scenario s (among BAU, FPS or NPS), as previously listed. $FC_{t-1,c}$ is the forest cover in country c at time $t-1$ in hectares.

Socio-economic scenarios

Within our socio-economic scenarios, we characterized more accurately the different drivers leading to deforestation. We assumed that deforestation within the Guiana Shield was mainly driven by three factors: gold-mining, agricultural and urban expansion. Following Hammond et al. (2007), gold production in the Guiana Shield was strongly correlated with gold prices. Assuming that deforestation due to gold-mining was proportional to gold production, we calibrated a model predicting the yearly intensity of deforestation due to gold-mining based on gold-prices (Dezécache et al., 2017). Deforestation due to gold-mining was extracted from binary deforestation maps using expert-based assessment of areas impacted by gold-mining (Debarros and Joubert, 2010; Rahm et al., 2015). Deforestation not occurring within gold-mining areas was assumed to be driven by agricultural and urban expansion.

We thus formulated a model of yearly deforestation within each country composed of two independent components, a gold-mining and a demographic component.

This model was formulated as follows:

$$Def_{t,c} = Def_{t,c}^{GM} + Def_{t,c}^{Dem}$$

with $Def_{t,c}$ corresponds to total predicted deforestation at year t in country c, which is the sum of predicted values of both gold-mining ($Def_{t,c}^{GM}$) and demographic ($Def_{t,c}^{Dem}$) components. Each component was formulated as follows:

$$Def_{t,c}^{GM} \; log\mathcal{N}(\theta_{0,c}^{GM} + \theta_c^V \times log(GoldPrice_t), \sigma^{GM})$$

$$Def_{t,c}^{Dem} \; log\mathcal{N}(\theta_0^{Dem} + \theta_1^{Dem} \times log(PopCh_c), \sigma^{Dem})$$

where θs are the models parameters, $GoldPrice_t$ is the yearly average international gold-price at year t, and $PopCh_c$ corresponds to yearly average predicted increase in population in country c.

We created two contrasted deforestation scenarios, differing in the value of gold, while the contribution of demography remains unchanged. In the low price scenario (GM-low), gold price corresponds to the average price over 2001-2014, whereas in the high price scenario (GM-high), it doubles the maximum price over the same period, thus corresponding to a value of 3077 USD/ounce. Although the volatility of gold prices makes it impossible to provide plausible future gold prices scenarios, these assumptions aimed at proposing an average and an extremely high (given past gold prices) case studies. From these models predicting yearly deforestation, a cumulated sum was calculated.

SM2 – List of geographical explanatory variables included in the deforestation location models

Table 1: *Shapefiles of protected areas and road network were provided respectively by Forest offices in Guyana (GFC), Suriname (SBB), French Guiana (ONF) and Amapa (IEF). **The shapefiles for Greenstone areas were manually digitized following the geological map produced by the Guyana Geology and Mines Commission (`http://www.ggmc.gov.gy/Documents/PDF/GeoServices/guy_geol.pdf`) in Guyana; obtained from the Surinamese forest office (SBB) in Suriname; obtained from French Geological Survey (BRGM) in French Guiana; and provided by the Scientific and Technological Research Institute (IEPA) in Amapá.

Variable name	Resolution (m)	Approx. Range	Sources
**Protected Areas**	30	Binary	*See legend
**Distance to nearest road**	150	0 – 170 km	**See legend
**Distance to nearest Greenstone area**	150	0 – 65 km	**See legend
**Distance to nearest stream following Strahler classification:**			Horton (1945); Strahler (1952); USGS (2000)
Order 1-3 (small)	150	0 – 2 km	
Order 4-6 (intermediate)	150	0 – 15 km	
Order 7 or + (large)	150	0 – 120 km	

SM3 – Future predicted deforestation maps (by increasing order of deforestation intensity)

HA

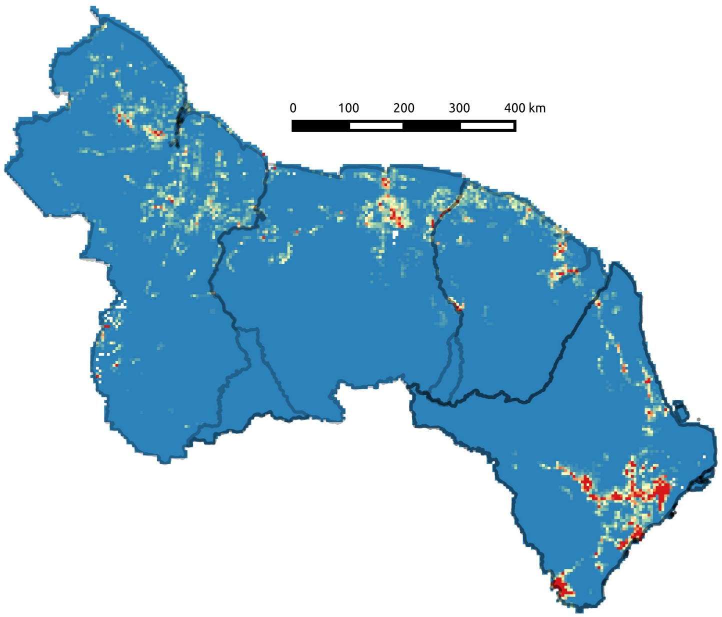

GM-LOW

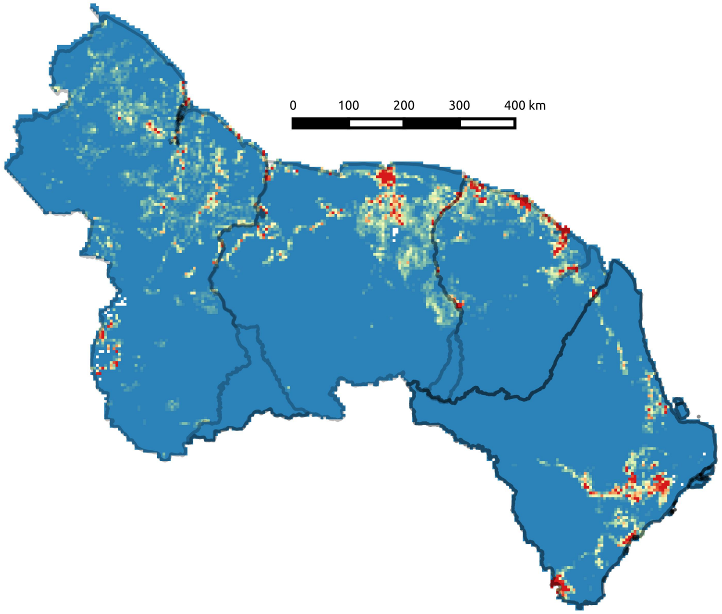

FPS-CI

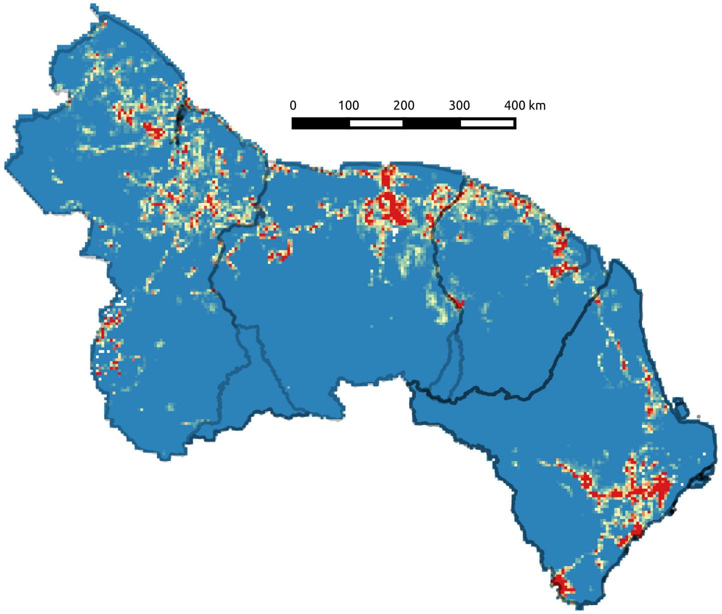

GM-HIGH

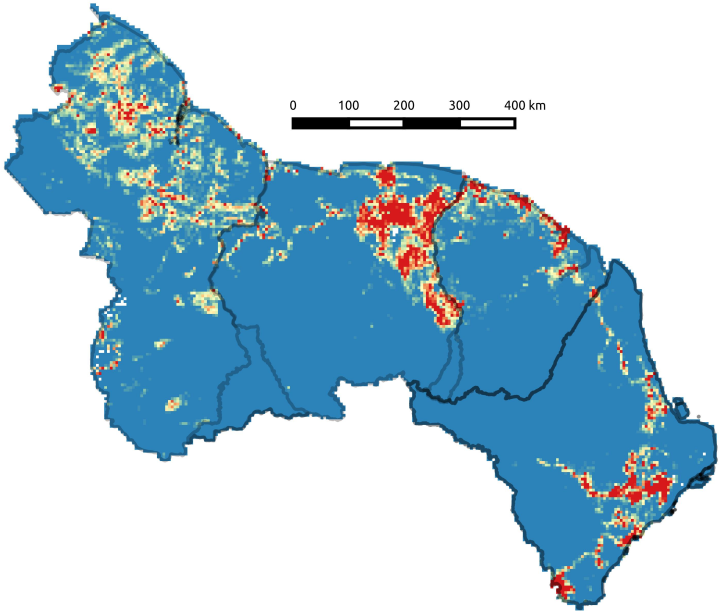

NPS-CI

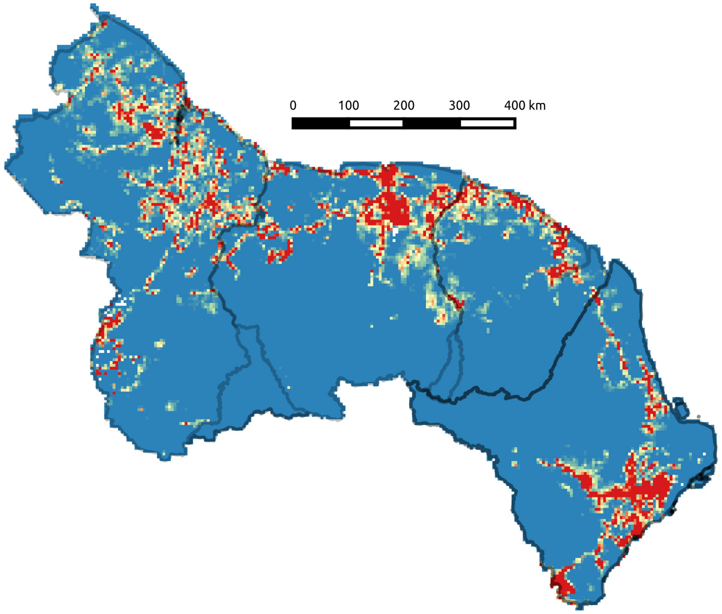

JRC2050

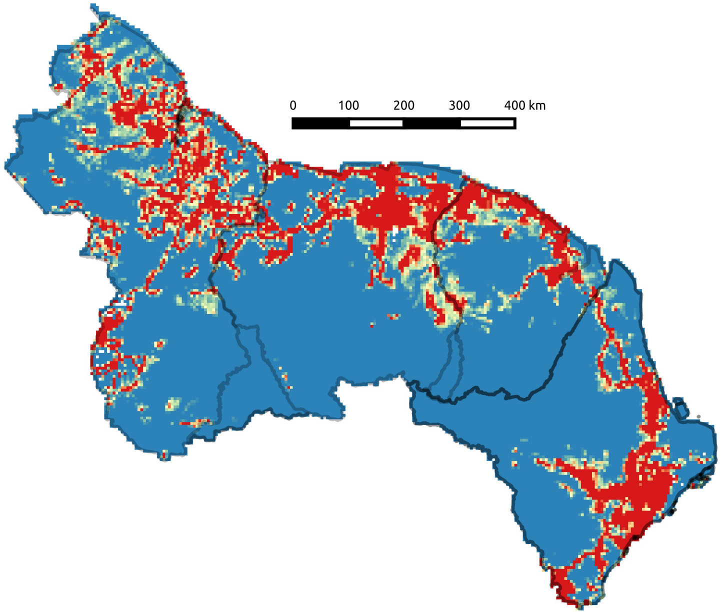

BAU-CI

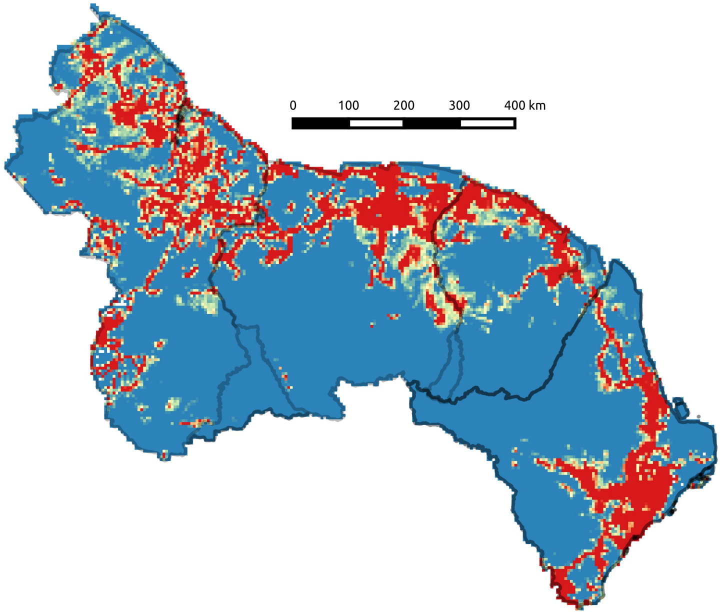

JRC2100

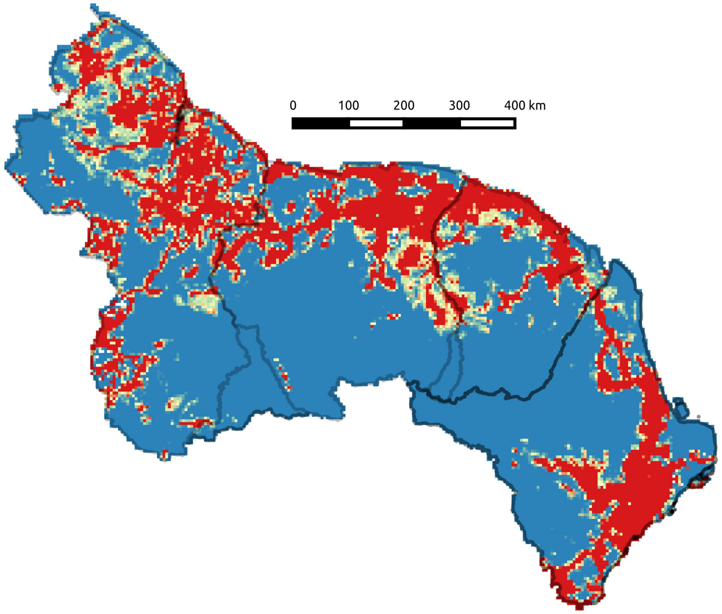

ERB

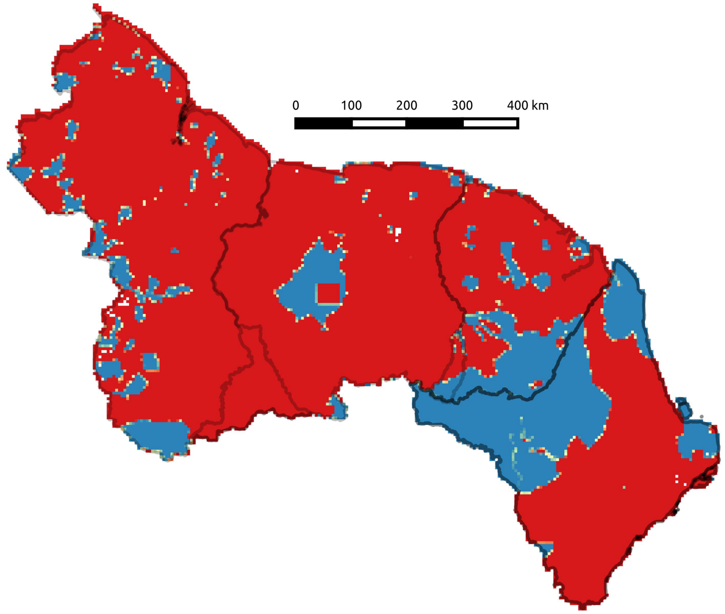

References

Angelsen, A., 2009. How do we set the reference levels for REDD payments ? Moving ahead with REDD (Cdm), 53–156.

Debarros, G., Joubert, P., 2010. Impact de l'activité aurifère sur le plateau des Guyanes. Rapport final. Tech. rep., ONF, Direction Régionale de Guyane, Cayenne.
URL http://d2ouvy59p0dg6k.cloudfront.net/downloads/2010{_}{_}etude{_}emprise{_}orpaillage{_}3{_}guyanes{_}finale.pdf

Dezécache, C., Faure, E., Gond, V., Salles, J.-M., Vieilledent, G., Hérault, B., 2017. Gold-rush in a forested El Dorado: deforestation leakages and the need for regional cooperation. Environmental Research Letters 12 (3), 034013.
URL http://stacks.iop.org/1748-9326/12/i=3/a=034013?key=crossref.f0dcd52ce7c53f4a44237cf42c7559e7

Hammond, D. S., Gond, V., de Thoisy, B., Forget, P.-M., DeDijn, B. P. E., 2007. Causes and consequences of a tropical forest gold rush in the Guiana Shield, South America. Ambio 36 (8), 661–670.

Horton, R. E., 1945. EROSIONAL DEVELOPMENT OF STREAMS AND THEIR DRAINAGE BASINS; HYDROPHYSICAL APPROACH TO QUANTITATIVE MORPHOLOGY. Geological Society Of America Bulletin 56, 275–370.

LCDS Guyana, 2011. Joint Concept Note.
URL https://www.regjeringen.no/globalassets/upload/md/2011/vedlegg/klima/klima{_}skogprosjektet/guyana/jointconceptnote{_}31mars2011.pdf

McKinsey & Company, 2008. Creating Incentives to Avoid Deforestation. Office (December), 35.

Mollicone, D., Achard, F., Federici, S., Eva, H. D., Grassi, G., Belward, A., Raes, F., Seufert, G., Stibig, H. J., Matteucci, G., Schulze, E. D., 2007. An incentive mechanism for reducing emissions from conversion of intact and non-intact forests. Climatic Change 83 (4), 477–493.

Rahm, M., Jullian, B., Lauger, A., de Carvalho, R., Vale, L., Totaram, J., Cort, K., Djojodikromo, M., Hardjoprajitno, M., Neri, S., Vieira, R., Watanabe, E., do Carmo Brito, M., Miranda, P., Paloeng, C., Moe Soe Let, V., Crabbe, S., Calmel, M., 2015. Monitoring the impact of gold mining on the forest cover and freshwater in the Guiana Shield. Reference year 2014. Tech. rep.

Strahler, A. N., 1952. Hypsometric (Area - Altitude) Analysis of Erosional Topography. Geological Society of America Bulletin 63 (11), 1117–1142.

USGS, 2000. Shuttle Radar Topography Mission (SRTM) 1 Arc-Second Global. Tech. rep.
